# Plasma phospholipid n-3 polyunsaturated fatty acids and major depressive disorder in Japanese elderly: the Japan Public Health Center-based Prospective Study

**DOI:** 10.1038/s41598-021-83478-5

**Published:** 2021-02-17

**Authors:** Kei Hamazaki, Yutaka J. Matsuoka, Taiki Yamaji, Norie Sawada, Masaru Mimura, Shoko Nozaki, Ryo Shikimoto, Shoichiro Tsugane

**Affiliations:** 1 Department of Public Health, Faculty of Medicine, University of Toyama, 2630 Sugitani, Toyama, Toyama, 930-0194 Japan; 2 Division of Health Care Research, Center for Public Health Sciences, National Cancer Center Japan, 5-1-1 Tsukiji, Chuo-ku, Tokyo, 104-0045 Japan; 3 Epidemiology and Prevention Group, Center for Public Health Sciences, National Cancer Center Japan, 5-1-1 Tsukiji, Chuo-ku, Tokyo, 104-0045 Japan; 4 Department of Neuropsychiatry, Keio University School of Medicine, 35 Shinanomachi, Shinjuku-ku, Tokyo, 160-8582 Japan

**Keywords:** Depression, Predictive markers

## Abstract

The beneficial effects of n-3 polyunsaturated fatty acids (PUFAs) such as eicosapentaenoic acid (EPA) and docosahexaenoic acid (DHA) on depression are not definitively known. In a previous population-based prospective cohort study, we found a reverse J-shaped association of intake of fish and docosapentaenoic acid (DPA), the intermediate metabolite of EPA and DHA, with major depressive disorder (MDD). To examine the association further in a cross-sectional manner, in the present study we analyzed the level of plasma phospholipid n-3 PUFAs and the risk of MDD in 1,213 participants aged 64–86 years (mean 72.9 years) who completed questionnaires and underwent medical check-ups, a mental health examination, and blood collection. In multivariate logistic regression analysis, odds ratios and 95% confidence intervals were calculated for MDD according to plasma phospholipid n-3 PUFA quartiles. MDD was diagnosed in 103 individuals. There were no significant differences in any n-3 PUFAs (i.e., EPA, DHA, or DPA) between individuals with and without MDD. Multivariate logistic regression analysis showed no significant association between any individual n-3 PUFAs and MDD risk. Overall, based on the results of this cross-sectional study, there appears to be no association of plasma phospholipid n-3 PUFAs with MDD risk in the elderly Japanese population.

## Introduction

It is reported that around 1% to 5% of the population aged 65 years or older are depressed and more than half of depressed older adults have late-life depression, that is, have a first episode after age 60^1^. Among the biological risks for late-life depression are cerebrovascular pathology, inflammation, endocrine status, and nutritional status^2^, and not only do medical illnesses increase the risk of late-life depression, depression itself predisposes to a variety of medical illnesses^3^. Among nutritional factors, a potential candidate for the prevention of depression is n-3 polyunsaturated fatty acids (PUFAs). It is suggested that four mechanisms might be involved in the association between n-3 PUFAs and depression, namely, neurotransmission, inflammation, oxidation, and neuroplasticity^4^. In terms of inflammation, metabolites derived from the n-3 PUFA eicosapentaenoic acid (EPA) are precursors for the anti-inflammatory series of eicosanoids, while among n-6 PUFAs, the counterparts of n-3 PUFAs, arachidonic acid is a known precursor of proinflammatory eicosanoids such as prostaglandin E2 and leukotriene B4^5^. Besides these anti-inflammatory eicosanoids, EPA and the n-3 PUFA docosahexaenoic acid (DHA) are metabolized to anti-inflammatory and pro-resolving mediators, including resolvins, protectins, and maresins^6^.

A recent meta-analysis of 26 randomized controlled trials (RCTs) involving a total of 2160 participants revealed that n-3 PUFAs with 60% or more of EPA at a dosage of ≤ 1 g/day would have beneficial effects on depression^7^. However, another meta-analysis of 9 RCTs among adults aged 60 years or older revealed no beneficial effects of n-3 PUFA supplementation on depression, although subgroup analysis showed a statistically significant effect at a dosage > 1.5 g/day^8^. In a population-based prospective cohort study, we previously examined whether there was any association of dietary fish and n-3 PUFA consumption with the risk of major depressive disorder (MDD) among elderly Japanese people^9^. We found no reduced risk of MDD in any quartiles for consumption of EPA or DHA but a reduced risk in the third quartile for consumption of fish and in the third quartile for consumption of docosapentaenoic acid (DPA), the intermediate metabolite of EPA and DHA. In this cohort study, we assessed consumption of fish (and n-3 PUFA intake) using a food frequency questionnaire (FFQ), which is dependent on participant recall and knowledge. A more objective method would be to measure specific fatty acids in the blood, the results of which would reflect tissue levels of n-3 PUFAs better than FFQ results or food records would^10^.

To our knowledge, four studies have examined the association between n-3 PUFA levels in the blood and the risk of depression among elderly community dwellers^11–14^. A cross-sectional study conducted in the Netherlands found generally small differences in individual PUFAs but a higher n-6/n-3 ratio in participants with depressive disorders compared with controls^11^. Another cross-sectional study conducted in France showed low EPA plasma concentrations associated with greater severity of depressive symptoms in older persons receiving antidepressant treatment^12^. Of two studies conducted in Japan, one cross-sectional study revealed inverse associations of serum EPA and DHA concentrations with depressive symptoms in Japanese middle-aged and elderly community dwellers^13^. The other cohort study (with a 5-year interval between collecting serum samples and assessing depression) found in the overall study population that the arachidonic acid (AA)/EPA ratio and AA/DHA ratio were not associated with the presence of depressive symptoms; however, in a subgroup with inflammatory findings, the AA/EPA ratio, but not the AA/DHA ratio, was associated with risk of depressive symptoms^14^.

Additional research of clinically diagnosed depression is warranted for two reasons. First, a meta-analysis reported larger differences in serum n-3 PUFA levels in studies that defined MDD according to the Diagnostic and Statistical Manual of Mental Disorders (DSM) compared with in studies not using the DSM criteria^15^. Second, a meta-analysis of RCTs revealed that n-3 PUFAs had a stronger effect on depressed mood in trials involving populations with major depression than in trials involving other populations^16^. To our knowledge, only one study has investigated a population that was clinically diagnosed with depression among community dwellers^11^, but this was the aforementioned study conducted in the Netherlands and not in Asian countries, such as Japan, where there is very high fish consumption, which is known to influence the blood n-3 PUFA level^17^.

Therefore, in this cross-sectional study involving elderly Japanese community dwellers (the same participants as in our previous prospective cohort study^9^), we investigated the association between plasma phospholipid levels of n-3 PUFAs and MDD risk.

## Methods

### Study population

This study was conducted as part of the Japan Public Health Center-based Prospective Study (JPHC Study), which is described in detail elsewhere^18^. In 2014–2015, we posted an invitation letter for mental health screening to 8,827 participants from the original 1990 cohort of 12,219 residents (6,172 men, 6047 women; age range 40–69 years) in the catchment area of Saku Public Health Center, Nagano Prefecture, after excluding those who had moved out of the study area, had died, or did not respond to the later questionnaires. Of the 1,299 who responded to the call for screening and who gave written informed consent to take part in this mental health screening, 1,279 completed the screening and provided a blood sample. After excluding 66 participants due to a diagnosis of dementia, 1,213 participants (516 men, 697 women) were included in the present analysis. We also conducted an exploratory analysis excluding those who had mild cognitive impairment (MCI; n = 779).

The JPHC study protocol was approved by the institutional review boards of the National Cancer Center Japan (2013-096) and the University of Toyama (R2016107). We conducted our research by adhering to the ethical principles outlined in the Declaration of Helsinki^19^.

### Assessment of current psychiatric and cognitive function

We administered the Center for Epidemiological Scale-Depression (CES-D)^20,21^ and the Patient Health Questionnaire-9 (PHQ-9)^22,23^ screening tests simultaneously at the mental health screening^9^. Then, each participant was assessed by a trained psychiatrist irrespective of their CES-D and PHQ-9 scores. CES-D and PHQ-9 scores were provided to the psychiatrist at the clinical interview. Finally, trained psychiatrists assessed whether the participants currently met the DSM-IV criteria for MDD^24^ after considering whether their depressive symptoms caused clinically significant distress or impairment. We did not assess the inter-rater reliability for the current major depressive episode.

Cognitive function was assessed by experienced neuropsychologists using the Mini-Mental State Examination (MMSE)^25^, Wechsler Memory Scale Revised (WMS-R) Logical Memory I and II subtests^26^, clock drawing test^27^, and Clinical Dementia Rating (CDR) Scale^28^. All participants were interviewed to confirm the number of years spent in formal education. We then categorized the participants’ cognitive function in accordance with criteria used in the Japan Alzheimer’s Disease Neuroimaging Initiative (J-ADNI)^29,30^, in which MCI was defined as amnestic MCI, as originally presented by Petersen et al^31^. Memory impairment was assessed by whether the participant’s score was below the education-adjusted cut-off level in the WMS-R Logical Memory II subtest (2 for 0–9 years, 4 for 10–15 years, and 8 for > 15 years). The MMSE cut-off point for dementia was set at 23/24. Finally, a trained psychiatrist determined the final diagnosis by combining the neuropsychological assessment, depressive symptom scales, and clinical interview findings. Diagnosis of dementia was made in accordance with the DSM-IV criteria. We diagnosed only dementia of all causes and did not classify the type of dementia. Participants who were not diagnosed as having dementia but were below the education-adjusted cut-off point for memory function were diagnosed as having MCI after their symptoms were confirmed by the psychiatrists.

### Blood collection and laboratory analysis

During the year of screening (2014–2015), non-fasting venous blood was drawn into vacutainer tubes containing ethylenediaminetetraacetic acid at baseline and plasma samples were extracted by centrifugation for 10 min at 1,200 × g and preserved at -80 °C until analysis. The fatty acid composition of the total phospholipid fraction was analyzed as described previously^32^. Briefly, total lipids were extracted from the plasma, the total phospholipid fraction was separated by thin-layer chromatography, and the fatty acids in the fraction were transmethylated and analyzed by gas chromatography (GC-2014; Shimadzu Corporation, Kyoto, Japan) with a DB-225 capillary column (length, 30 m; internal diameter, 0.25 mm; film, 0.25 μm; J&M Scientific, Folsom, CA). The system was controlled using the gas chromatographic software GCsolution, version 2.3 (Shimadzu Corporation). The inter-assay coefficients of variance were 2.0% for EPA and 2.1% for DHA. Fatty acids were expressed as the area percentage of total fatty acids. To avoid bias, fatty acid measurements were performed by laboratory personnel who were blinded to MDD status.

### Statistical analysis

Data are expressed as the mean ± standard deviation or median unless stated otherwise. The chi-square test was used for categorical variables and the t-test was used for continuous variables. After individuals without MDD were categorized according to the quartile distributions of plasma phospholipid fatty acid level using the SAS program, boundary values were calculated by averaging the maximum and minimum values of lower and higher quartiles, respectively. For example, the first boundary value was calculated by averaging the maximum value of first quartile and minimum value of the second quartile. Although the original values (first, second and third boundaries) were computed to seven decimal places, the respective values shown are rounded to one or two decimal places: 9.8, 11.2, and 12.9 for total n-3 PUFAs; 0.29, 0.33, and 0.39 for α-linolenic acid (ALA); 1.93, 2.63, and 3.56 for EPA; 6.40, 7.22, and 7.99 for DHA; 0.82, 0.95, and 1.11 for DPA; 27.5, 29.4, and 31.0 for total n-6 PUFAs; 17.1, 19.2, and 21.1 for linoleic acid (LA); 6.70, 7.47, and 8.33 for AA; 2.16, 2.59, and 3.12 for n-6/n-3 ratio; 2.01, 2.86, and 4.05 for AA/EPA ratio; and 0.89, 1.04, and 1.23 for AA/DHA ratio. Then, logistic regression analysis was used to calculate odds ratios (ORs) and 95% confidence intervals (CIs) for current MDD diagnosis compared with the lowest category as reference. The multivariate model was then adjusted for the following potential confounding variables identified from questionnaire responses (2014–2015): age, sex, history of depression (yes or no), history of cancer (yes or no), history of stroke (yes or no), history of myocardial infarction (yes or no), and history of diabetes mellitus (yes or no). We tested trends across quartiles for total n-3 PUFAs, ALA, EPA, DHA, DPA, total n-6 PUFAs, LA, AA, n-3/n-6 ratio, AA/EPA ratio, and AA/DHA ratio using ordinal numbers (0–3) assigned to quartile categories. All *P* values are two-sided and statistical significance was set at *P* < 0.05. All statistical analysis was performed with SAS software version 9.4 (SAS Institute, Inc., Cary, NC).

## Results

In total, 103 participants were diagnosed with MDD by a trained psychiatrist. Table 1 shows the characteristics of these 103 individuals with MDD and the remaining 1,110 without MDD. The individuals with MDD were older than those without MDD and were more likely to have a history of depression. There were no significant differences in the history of other diseases such as diabetes, cancer, stroke, or myocardial infarction.

**Table 1:** Participant characteristics.

	Individuals with MDD	Individuals without MDD	*P* value
	(n = 103)	(n = 1,110)	
Age, years ± SD	74.1 ± 5.5	72.8 ± 5.5	0.02
Male (%)	35.9	43.2	0.16
History of depression (yes), %	6.8	1.7	0.0007
History of diabetes (yes), %	9.7	9.9	0.95
History of cancer (yes), %	13.6	12.4	0.73
History of stroke (yes), %	1.0	4.3	0.098
History of myocardial infarction (yes), %	1.9	1.7	0.86
Prevalence of mild cognitive impairment, n (%)	44 (43)	390 (35)	0.12

The chi-square test was used for categorical variables and the t-test was used for continuous variables. A *p* value less than 0.05 was considered statistically significant.

Abbreviation: MDD, major depressive disorder.

Table 2 shows the plasma phospholipid fatty acid compositions of the individuals with and without MDD. There were no significant differences between the two groups in individual saturated fatty acids, monounsaturated fatty acids, n-3 PUFAs, or the n-3/n-6 ratio. Only AA was significantly higher in individuals with MDD than in those without MDD (percentage of total fatty acids: 7.92% vs. 7.60%; p = 0.02).

**Table 2:** Plasma fatty acid compositions of individuals with and without MDD.

		Individuals with MDD	Individuals without MDD	*P* value
		(n = 103)	(n = 1,110)	
Fatty acids	Structure	Percentage of total fatty acids		
**Saturated fatty acids**				
Myristic acid	14:0	0.33 ± 0.07	0.32 ± 0.06	0.36
Palmitic acid	16:0	28.49 ± 1.31	28.53 ± 1.33	0.80
Stearic acid	18:0	14.52 ± 0.97	14.54 ± 1.02	0.90
Arachidic acid	20:0	0.39 ± 0.07	0.39 ± 0.07	0.79
Behenic acid	22:0	0.94 ± 0.19	0.97 ± 0.20	0.11
Lignoceric acid	24:0	0.88 ± 0.18	0.90 ± 0.18	0.22
Total saturated fatty acids		45.55 ± 1.02	45.65 ± 1.10	0.38
**Monounsaturated fatty acids**				
Palmitoleic acid	16:1 n-7	0.54 ± 0.17	0.54 ± 0.17	0.92
Vaccenic acid	18:1 n-7	1.61 ± 0.25	1.58 ± 0.23	0.22
Oleic acid	18:1 n-9	9.48 ± 1.28	9.47 ± 1.23	0.94
Gondoic acid	20:1 n-9	0.19 ± 0.06	0.20 ± 0.07	0.34
Nervonic acid	24:1 n-9	1.86 ± 0.40	1.89 ± 0.41	0.48
Total monounsaturated fatty acids		13.69 ± 1.47	13.69 ± 1.39	0.98
**n-6 PUFAs**				
Linoleic acid	18:2 n-6	18.92 ± 2.66	19.06 ± 2.83	0.62
γ-Linoleic acid	18:3 n-6	0.08 ± 0.04	0.08 ± 0.04	0.12
Eicosadienoic acid	20:2 n-6	0.29 ± 0.05	0.29 ± 0.05	0.59
Dihomo-γ-linolenic acid	20:3 n-6	2.03 ± 0.55	1.98 ± 0.52	0.34
AA	20:4 n-6	7.92 ± 1.60	7.60 ± 1.34	0.02
Docosatetraenoic acid	22:4 n-6	0.15 ± 0.06	0.15 ± 0.10	0.48
Total n-6 polyunsaturated fatty acids		29.39 ± 2.41	29.16 ± 2.73	0.42
**n-3 PUFAs**				
α-Linolenic acid	18:3 n-3	0.35 ± 0.10	0.35 ± 0.10	0.81
EPA	20:5 n-3	2.75 ± 1.19	2.92 ± 1.36	0.23
DPA	22:5 n-3	0.98 ± 0.21	0.99 ± 0.25	0.96
DHA	22:6 n-3	7.29 ± 1.19	7.25 ± 1.28	0.77
Total n-3 polyunsaturated fatty acids		11.37 ± 2.10	11.50 ± 2.44	0.60
n-6/n-3 ratio		2.71 ± 0.71	2.69 ± 0.79	0.84
AA/EPA		3.49 ± 1.96	3.22 ± 1.72	0.13
AA/DHA		1.12 ± 0.33	1.09 ± 0.30	0.22

Values are means ± SD.

Abbreviations: AA, arachidonic acid; DHA, docosahexaenoic acid; DPA, docosapentaenoic acid; EPA, eicosapentaenoic acid; MDD, major depressive disorder; PUFAs, polyunsaturated fatty acids.

Total saturated fatty acids: 14:0 + 16:0 + 18:0 + 20:0 + 22:0 + 24:0

Total monounsaturated fatty acids: 16 : 1 n-7 + 18 : 1 n-7 + 18 : 1 n-9 + 20 : 1 n-9 + 24 : 1 n-9.

Total n-6 polyunsaturated fatty acids: 18 : 2 n-6 + 18 : 3 n-6 + 20 : 2 n-6 + 20 : 3 n-6 + 20 : 4 n-6 + 22 : 4 n-6.

Total n-3 polyunsaturated fatty acids: 18 : 3 n-3 + 20 : 5 n-3 + 22 : 5 n-3 + 22 : 6 n-3.

Table 3 presents the ORs and 95% CIs for MDD according to quartile of plasma phospholipid PUFAs. There was no reduced risk of MDD in any quartile for individual n-3 or n-6 PUFAs, but trend tests revealed marginal significance for AA (p for trend = 0.08): the higher the AA, the higher the risk. There were no reduced risks of MDD in any quartile for either total monounsaturated fatty acids or total saturated fatty acids (data not shown).

**Table 3:** Odds ratios and 95% confidence intervals for MDD according to quartile of plasma PUFAs in the JPHC Study (n = 1,213).

	Quartile of fatty acids				*P* for trend
	1 (low)	2	3	4 (high)	
Total n-3 PUFAs (median for controls, %)	8.9	10.6	12.0	14.4	
Individuals with MDD	27	21	31	24	
Individuals without MDD	277	278	278	277	
Model 1^a^	1.00	0.78 (0.48–1.40)	1.14 (0.67–1.97)	0.89 (0.50–1.58)	0.97
Model 2^b^	1.00	0.76 (0.41–1.38)	1.19 (0.68–2.06)	0.87 (0.49–1.56)	0.96
α–Linolenic acid (median for controls, %)	0.26	0.31	0.36	0.45	
Individuals with MDD	26	23	29	25	
Individuals without MDD	277	278	278	277	
Model 1^a^	1.00	0.88 (0.49–1.58)	1.11 (0.62–1.94)	0.96 (0.54–1.71)	0.90
Model 2^b^	1.00	0.90 (0.50–1.63)	1.11 (0.63–1.96)	0.92 (0.51–1.66)	0.98
EPA (median for controls, %)	1.59	2.25	3.07	4.55	
Individuals with MDD	26	27	30	20	
Individuals without MDD	277	278	278	277	
Model 1^a^	1.00	1.03 (0.59–1.82)	1.15 (0.66–1.99)	0.77 (0.42–1.41)	0.53
Model 2^b^	1.00	1.00 (0.57–1.78)	1.21 (0.69–2.12)	0.80 (0.43–1.48)	0.68
DHA (median for controls, %)	5.79	6.84	7.58	8.68	
Individuals with MDD	22	29	25	27	
Individuals without MDD	277	278	278	277	
Model 1^a^	1.00	1.31 (0.74–2.34)	1.13 (0.62–2.06)	1.23 (0.68–2.21)	0.64
Model 2^b^	1.00	1.31 (0.73–2.35)	1.12 (0.61–2.05)	1.16 (0.64–2.10)	0.79
DPA (median for controls, %)	0.73	0.89	1.02	1.27	
Individuals with MDD	23	23	35	22	
Individuals without MDD	277	278	278	277	
Model 1^a^	1.00	1.00 (0.55–1.82)	1.52 (0.87–2.63)	0.96 (0.52–1.76)	0.70
Model 2^b^	1.00	1.01 (0.55–1.85)	1.50 (0.86–2.63)	0.97 (0.53–1.80)	0.67
Total n-6 PUFAs (median for controls, %)	25.9	28.5	30.2	32.1	
Individuals with MDD	19	28	29	27	
Individuals without MDD	277	278	278	277	
Model 1^a^	1.00	1.47 (0.80–2.69)	1.52 (0.83–2.78)	1.42 (0.77–2.62)	0.29
Model 2^b^	1.00	1.53 (0.83–2.82)	1.54 (0.84–2.84)	1.37 (0.73–2.55)	0.38
Linoleic acid (median for controls, %)	15.6	18.2	20.1	22.2	
Individuals with MDD	27	25	31	20	
Individuals without MDD	277	278	278	277	
Model 1^a^	1.00	0.92 (0.52–1.63)	1.14 (0.67–1.97)	0.74 (0.41–1.35)	0.53
Model 2^b^	1.00	1.03 (0.58–1.82)	1.14 (0.66–1.97)	0.75 (0.41–1.38)	0.49
AA (median for controls, %)	6.17	7.08	7.84	9.12	
Individuals with MDD	26	15	26	36	
Individuals without MDD	277	278	278	277	
Model 1^a^	1.00	0.57 (0.30–1.11)	1.00 (0.56–1.76)	1.38 (0.81–2.36)	0.09
Model 2^b^	1.00	0.55 (0.28–1.07)	1.01 (0.57–1.80)	1.40 (0.81–2.40)	0.08
n-6/n-3 ratio (median for controls)	1.85	2.39	2.83	3.59	
Individuals with MDD	23	32	19	29	
Individuals without MDD	280	271	285	274	
Model 1^a^	1.00	1.44 (0.82–2.52)	0.81 (0.43–1.52)	1.29 (0.73–2.28)	0.82
Model 2^b^	1.00	1.48 (0.84–2.62)	0.79 (0.42–1.49)	1.30 (0.73–2.32)	0.85
AA/EPA (median for controls)	1.57	2.46	3.32	4.90	
Individuals with MDD	19	28	30	26	
Individuals without MDD	277	278	278	277	
Model 1^a^	1.00	1.47 (0.80–2.69)	1.57 (0.86–2.86)	1.37 (0.74–2.53)	0.33
Model 2^b^	1.00	1.46 (0.79–2.70)	1.53 (0.83–2.80)	1.34 (0.72–2.50)	0.39
AA/DHA (median for controls)	0.79	0.96	1.12	1.43	
Individuals with MDD	24	20	25	34	
Individuals without MDD	277	278	278	277	
Model 1^a^	1.00	0.83 (0.45–1.54)	1.04 (0.58–1.86)	1.42 (0.82–2.45)	0.14
Model 2^b^	1.00	0.84 (0.45–1.57)	1.01 (0.56–1.82)	1.46 (0.83–2.54)	0.14

Fatty acids are expressed as percentage of total fatty acid.

Abbreviations: AA, arachidonic acid; DHA, docosahexaenoic acid; DPA, docosapentaenoic acid; EPA, eicosapentaenoic acid; MDD, major depressive disorder; PUFAs, polyunsaturated fatty acids.

^a^ Crude odds ratios.

^b^ Adjusted for age, sex, past history of depression, cancer, stroke, myocardial infarction, and diabetes mellitus.

After individuals without MDD were categorized according to the quartile distributions of plasma phospholipid fatty acid level using the SAS program, boundary values were calculated by averaging the maximum and minimum values of lower and higher quartiles, respectively. For example, the first boundary value was calculated by averaging the maximum value of first quartile and minimum value of the second quartile. Although the original values (first, second and third boundaries) were computed to seven decimal places, the respective values shown are rounded to one or two decimal places: 9.8, 11.2, and 12.9 for total n-3 PUFAs; 0.29, 0.33, and 0.39 for α-linolenic acid (ALA); 1.93, 2.63, and 3.56 for EPA; 6.40, 7.22, and 7.99 for DHA; 0.82, 0.95, and 1.11 for DPA; 27.5, 29.4, and 31.0 for total n-6 PUFAs; 17.1, 19.2, and 21.1 for linoleic acid (LA); 6.70, 7.47, and 8.33 for AA; 2.16, 2.59, and 3.12 for n-6/n-3 ratio; 2.01, 2.86, and 4.05 for AA/EPA ratio; and 0.89, 1.04, and 1.23 for AA/DHA ratio.

The exploratory analysis involving individuals without MCI (n = 779) revealed that there was no significant risk reduction for any individual quartile. However, trend tests showed significant positive (detrimental) associations of MDD risk with AA (p = 0.03) and AA/DHA (p = 0.04) but not with other fatty acids.

## Discussion

To our knowledge, this is the first study to examine the association between plasma phospholipid n-3 PUFAs and the risk of MDD in Japanese older community dwellers. We found no reduced MDD risk for any of the individual n-3 or n-6 PUFAs or for the n-3/n-6 ratio. Only a marginal association (p = 0.08) was found between AA and increased risk of MDD. In our previous cohort study using the same population, we found that reduced MDD risk was associated with consumption of fish and DPA, but not with any quartiles for consumption of EPA and DHA^9^. The notable differences between the two studies are in their design (longitudinal vs. cross-sectional) and in the analysis of PUFAs (FFQ vs. tissue levels). The advantage of measuring tissue PUFA levels is its potential to be more quantitative than with the FFQ^33^. The discrepancies between our previous and present studies might be due to nutrients in fish other than n-3 PUFAs (i.e., vitamins, minerals, and calcium). Dietary intake of n-3 PUFAs, especially long-chain ones (EPA, DPA, and DHA), was mostly from fish. Fish is also a good source of vitamin D, and a beneficial effect of vitamin D on depression in older adults was reported in a meta-analysis of cohort studies^43^. A cross-sectional study of an elderly population in Japan found that dietary calcium intake was significantly and negatively correlated with depressive symptoms among women but not among men^34^, although a meta-analysis of 17 epidemiological studies reported in 12 articles found no significant association between dietary calcium intake and risk of depression^35^. A cohort study of an elderly population in rural China showed that higher selenium levels were associated with less severe depressive symptoms, although the association was no longer significant after adjusting for cognition^36^. Taken together, the beneficial effects of fish consumption on the risk of MDD that we found in our previous study^9^ might also be attributed to other nutrients (vitamin D, selenium, calcium, etc.) rather than to n-3 PUFAs only.

The other possible reason for the discrepancies might be that fatty acid tissue levels reflect dietary fatty acid intake for only a short time. Although measuring blood level of fatty acids is objective^33^ and has a relatively high correlation with n-3 PUFAs of marine origin^37^, these biomarkers reflect intake over the preceding few weeks only, not over the long term^33^. On the other hand, the FFQ reflected dietary intake over at least the past year. If late-life depression is a consequence of biological risk factors such as cerebrovascular pathology, inflammation, endocrine status, and nutritional status^2^, meaning that late-life depression may be established over a long period of time, we can reasonably suppose that the FFQ has a stronger association than fatty acid tissue levels with the risk of MDD.

A similar cross-sectional study to ours that was also conducted in Japan found an inverse association between blood levels of EPA and DHA and the risk of depressive symptoms^13^. The main difference between the two studies was the age of the participants when they were assessed: 73.3 years in our study versus 60.3 years in the other study. Although both studies excluded individuals diagnosed with dementia, our study might have had more participants with MCI. In fact, in our exploratory analysis excluding individuals with MCI, significant positive (detrimental) associations of MDD risk with AA and AA/DHA emerged. From these results, we speculate that some PUFAs that are not involved in MCI might be associated with MDD.

One of the major reasons we did not find associations between plasma phospholipid n-3 PUFAs and MDD risk might be due to the ceiling effect of n-3 PUFA intake. In fact, if we look closely at the difference in mean baseline intake of n-3 PUFAs between the present study^9^ and the abovementioned Japanese study^13^, it is about 0.5 g/day (3.0 g/day vs. 2.5 g/day^38^, respectively). Individuals with lower dietary intake of n-3 PUFAs are much more likely to see clinical benefits of additional n-3 PUFA intake than those with higher intake^39^. Our exploratory findings are also similar to those from a previous cohort study of Japanese community dwellers^14^, which found that AA/EPA but not AA/DHA was associated with the risk of depressive symptoms in a subgroup with inflammatory findings (CRP ≥ 1.0 mg/L). Although we do not have a clear answer as to why we found an association with AA/DHA but not AA/EPA, the underlying mechanisms are essentially based on the proinflammatory and anti-inflammatory properties of n-6 and n-3 PUFAs, respectively^5^. Further study is needed to clarify the role of inflammatory findings in these associations.

Brain imaging is another potential area of future research. Blood levels of n-3 fatty acids are known to be inversely associated with intima-media thickness of the carotid artery (reflecting cerebrovascular atherosclerosis) in Japanese men^40^. Given that cerebrovascular disease is closely associated with vascular depression^41^, brain imaging modalities such as magnetic resonance imaging might shed light on the underlying mechanism of the effects of n-3 PUFAs on late-onset depression.

One of the strengths of this study is that we objectively measured specific fatty acids in the blood, which better reflects tissue levels of n-3 PUFAs than the more subjective measures of the FFQ and food records^10^. Another strength is that we analyzed data from a large population of Japanese community dwellers and MDD was diagnosed based on DSM criteria. Most of the studies on MDD and n-3 PUFAs have been conducted in hospital settings (case–control studies) with rather small numbers^15^, except for the aforementioned cross-sectional study conducted in the Netherlands^11^. Comparisons based on case–control studies are likely to be biased when controls are selected from ill-defined study bases and thus do not adequately reflect the exposure experience of the true source population^42^. Another advantage of the present study is that the data analyzed were obtained from repeated use of the same questionnaire.

The main limitation of this study is that its cross-sectional design precludes evaluation of causal relationships. Also, data on lifestyle factors such as smoking, alcohol intake, and physical activity were not collected, brain imaging (e.g. magnetic resonance imaging) was not performed, and inflammatory biomarkers (e.g. C-reactive protein) were not measured. There may also have been selection bias because only 14% of Saku residents participated in the 2014–2015 mental health screening. The only way to avoid selection bias is to conduct an RCT. Lastly, our findings were obtained from a population with very high fish consumption, so they will not necessarily apply to all nationalities and ethnicities.

In conclusion, this population-based cross-sectional study examining the association between plasma phospholipid n-3 PUFAs and MDD risk in an elderly Japanese population revealed no reduced risk for any of the individual n-3 or n-6 PUFAs or for the n-3/n-6 ratio. However, there was a marginal association between AA and increased risk of MDD. Further study is warranted, especially in the form of RCTs, to clarify the efficacy of n-3 PUFAs for MDD in the elderly.

## References

[1] Fiske A, Wetherell JL, Gatz M (2009). Depression in older adults. Annu. Rev. Clin. Psychol..

[2] Tiemeier H (2003). Biological risk factors for late life depression. Eur. J. Epidemiol..

[3] Alexopoulos GS (2019). Mechanisms and treatment of late-life depression. Transl. Psychiatry.

[4] Su KP, Matsuoka Y, Pae CU (2015). Omega-3 polyunsaturated fatty acids in prevention of mood and anxiety disorders. Clin. Psychopharmacol. Neurosci..

[5] Simopoulos AP (2002). Omega-3 fatty acids in inflammation and autoimmune diseases. J. Am. Coll. Nutr..

[6] Calder PC (2015). Marine omega-3 fatty acids and inflammatory processes: effects, mechanisms and clinical relevance. Biochim. Biophys. Acta.

[7] Liao Y (2019). Efficacy of omega-3 PUFAs in depression: a meta-analysis. Transl Psychiatry.

[8] Bai ZG, Bo A, Wu SJ, Gai QY, Chi I (2018). Omega-3 polyunsaturated fatty acids and reduction of depressive symptoms in older adults: a systematic review and meta-analysis. J. Affect. Disord..

[9] Matsuoka YJ (2017). Dietary fish, n-3 polyunsaturated fatty acid consumption, and depression risk in Japan: a population-based prospective cohort study. Transl. Psychiatry.

[10] de Dahl L (2011). A short food frequency questionnaire to assess intake of seafood and n-3 supplements: validation with biomarkers. Nutr. J..

[11] Tiemeier H, van Tuijl HR, Hofman A, Kiliaan AJ, Breteler MM (2003). Plasma fatty acid composition and depression are associated in the elderly: the Rotterdam Study. Am. J. Clin. Nutr..

[12] Feart C (2008). Plasma eicosapentaenoic acid is inversely associated with severity of depressive symptomatology in the elderly: data from the Bordeaux sample of the Three-City Study. Am. J. Clin. Nutr..

[13] Horikawa C (2016). Cross-sectional association between serum concentrations of n-3 long-chain PUFA and depressive symptoms: results in Japanese community dwellers. Br. J. Nutr..

[14] Shibata M (2018). Association between the ratio of serum arachidonic acid to eicosapentaenoic acid and the presence of depressive symptoms in a general Japanese population: the Hisayama Study. J. Affect. Disord..

[15] Lin PY, Huang SY, Su KP (2010). A meta-analytic review of polyunsaturated fatty acid compositions in patients with depression. Biol. Psychiatry.

[16] Appleton KM (2006). Effects of n-3 long-chain polyunsaturated fatty acids on depressed mood: systematic review of published trials. Am. J. Clin. Nutr..

[17] Yamada T (2000). Atherosclerosis and omega-3 fatty acids in the populations of a fishing village and a farming village in Japan. Atherosclerosis.

[18] Tsugane S, Sawada N (2014). The JPHC study: design and some findings on the typical Japanese diet. Jpn. J. Clin. Oncol..

[19] General Assembly of the World Medical Association (2014). World Medical Association Declaration of Helsinki: ethical principles for medical research involving human subjects. J. Am. Coll. Dent..

[20] Radloff LS (1977). The CES-D scale: a self-report depression scale for research in the general population. Appl. Psychol. Meas..

[21] Shima S, Shikano T, Kitamura T (1985). A new self-report depression scale (in Japanese). Seishinigaku.

[22] Kroenke K, Spitzer RL, Williams JB (2001). The PHQ-9: validity of a brief depression severity measure. J. Gen. Intern. Med..

[23] Muramatsu K (2007). The patient health questionnaire, Japanese version: validity according to the mini-international neuropsychiatric interview-plus. Psychol. Rep..

[24] American Psychiatric Association (1994). Diagnostic Criteria from DSM-IV.

[25] Folstein MF, Folstein SE, McHugh PR (1975). "Mini-mental state". A practical method for grading the cognitive state of patients for the clinician. J. Psychiatr. Res..

[26] Elwood RW (1991). The Wechsler Memory Scale-Revised: psychometric characteristics and clinical application. Neuropsychol. Rev..

[27] Agrell B, Dehlin O (1998). The clock-drawing test. Age Ageing.

[28] Hughes CP, Berg L, Danziger WL, Coben LA, Martin RL (1982). A new clinical scale for the staging of dementia. Br. J. Psychiatry.

[29] Fujishima M (2017). Sample size estimation for Alzheimer's disease trials from Japanese ADNI serial magnetic resonance imaging. J. Alzheimers Dis..

[30] Iwatsubo T (2010). Japanese Alzheimer's Disease Neuroimaging Initiative: present status and future. Alzheimers Dement.

[31] Petersen RC (1999). Mild cognitive impairment: clinical characterization and outcome. Arch. Neurol..

[32] Hamazaki K (2018). Plasma levels of n-3 fatty acids and risk of coronary heart disease among Japanese: the Japan Public Health Center-based (JPHC) study. Atherosclerosis.

[33] Hodson L, Skeaff CM, Fielding BA (2008). Fatty acid composition of adipose tissue and blood in humans and its use as a biomarker of dietary intake. Prog. Lipid Res..

[34] Thi Thu Nguyen T (2019). Association between lower intake of minerals and depressive symptoms among elderly Japanese women but not men: findings from Shika study. Nutrients.

[35] Li B, Lv J, Wang W, Zhang D (2017). Dietary magnesium and calcium intake and risk of depression in the general population: a meta-analysis. Aust. N. Z. J. Psychiatry.

[36] Gao S (2012). Selenium level and depressive symptoms in a rural elderly Chinese cohort. BMC Psychiatry.

[37] Kobayashi M, Sasaki S, Kawabata T, Hasegawa K, Tsugane S (2003). Validity of a self-administered food frequency questionnaire used in the 5-year follow-up survey of the JPHC Study Cohort I to assess fatty acid intake: comparison with dietary records and serum phospholipid level. J. Epidemiol..

[38] Horikawa C (2018). Longitudinal association between n-3 long-chain polyunsaturated fatty acid intake and depressive symptoms: a population-based cohort study in Japan. Nutrients.

[39] Mozaffarian D, Rimm EB (2006). Fish intake, contaminants, and human health: evaluating the risks and the benefits. JAMA.

[40] Sekikawa A (2008). Marine-derived n-3 fatty acids and atherosclerosis in Japanese, Japanese-American, and white men: a cross-sectional study. J. Am. Coll. Cardiol..

[41] Alexopoulos GS, Bruce ML, Silbersweig D, Kalayam B, Stern E (1999). Vascular depression: a new view of late-onset depression. Dialogues Clin. Neurosci..

[42] Lunet N, Azevedo A (2009). On the comparability of population-based and hospital-based case-control studies. Gac. Sanit..

[43] Li, H. *et al.* Serum 25-Hydroxyvitamin D levels and depression in older adults: a rose–response meta-analysis of prospective cohort studies. *Am. J. Geriat. Psychiat.* **27**(11), 1192–1202 (2019). 10.1016/j.jagp.2019.05.022 31262683

